# Evaluation of the Health-related Quality of Life of Children in *Schistosoma haematobium*-endemic Communities in Kenya: A Cross-sectional Study

**DOI:** 10.1371/journal.pntd.0002106

**Published:** 2013-03-07

**Authors:** Carolyn C. Terer, Amaya L. Bustinduy, Ruth V. Magtanong, Ng'ethe Muhoho, Peter L. Mungai, Eric M. Muchiri, Uriel Kitron, Charles H. King, Francis M. Mutuku

**Affiliations:** 1 Department of Public Health, School of Health Sciences, Kenyatta University, Nairobi, Kenya; 2 Center for Global Health and Diseases, Case Western Reserve University, Cleveland, Ohio, United States of America; 3 Department of Anthropology, Case Western Reserve University, Cleveland, Ohio, United States of America; 4 Department of Pathology, School of Health Sciences, Kenyatta University, Nairobi, Kenya; 5 Division of Vector Borne and Neglected Tropical Diseases, Ministry of Public Health and Sanitation, Nairobi, Kenya; 6 Department of Environmental Studies, Emory University, Atlanta, Georgia, United States of America; Ministère de la Santé Publique et de la Lutte contre les Endémies, Niger

## Abstract

**Background:**

Schistosomiasis remains a global public health challenge, with 93% of the ∼237 million infections occurring in sub-Saharan Africa. Though rarely fatal, its recurring nature makes it a lifetime disorder with significant chronic health burdens. Much of its negative health impact is due to non-specific conditions such as anemia, undernutrition, pain, exercise intolerance, poor school performance, and decreased work capacity. This makes it difficult to estimate the disease burden specific to schistosomiasis using the standard DALY metric.

**Methodology/Principal Findings:**

In our study, we used Pediatric Quality of Life Inventory (PedsQL), a modular instrument available for ages 2–18 years, to assess health-related quality of life (HrQoL) among children living in a *Schistosoma haematobium*-endemic area in coastal Kenya. The PedsQL questionnaires were administered by interview to children aged 5–18 years (and their parents) in five villages spread across three districts. HrQoL (total score) was significantly lower in villages with high prevalence of *S. haematobium* (−4.0%, p<0.001) and among the lower socioeconomic quartiles (−2.0%, p<0.05). A greater effect was seen in the psychosocial scales as compared to the physical function scale. In moderate prevalence villages, detection of any parasite eggs in the urine was associated with a significant 2.1% (p<0.05) reduction in total score. The PedsQL reliabilities were generally high (Cronbach alphas ≥0.70), floor effects were acceptable, and identification of children from low socioeconomic standing was valid.

**Conclusions/Significance:**

We conclude that exposure to urogenital schistosomiasis is associated with a 2–4% reduction in HrQoL. Further research is warranted to determine the reproducibility and responsiveness properties of QoL testing in relation to schistosomiasis. We anticipate that a case definition based on more sensitive parasitological diagnosis among younger children will better define the immediate and long-term HrQoL impact of *Schistosoma* infection.

## Author Summary

Because urogenital schistosomiasis is a multi-decadal chronic disease that begins in early childhood, and because it is a disease that may affect nearly everyone in endemic communities, its impact on personal health-related quality of life (HrQoL) has been difficult to gauge accurately. In order to provide a more precise estimate of schistosomiasis' impact on overall health status, we used a standardized questionnaire, the PedsQL SF15 Inventory, to interview children, aged 5–18 years, and their parents, to quantify their reported physical, social, emotional, and scholastic performance status. Scores were significantly lower in villages having high *Schistosoma* prevalence, as compared those having moderate prevalence. In adjusting for age, sex, socioeconomic standing, undernutrition, anemia, and hookworm parasites, we found that relative poverty, stunting, wasting, and *S. haematobium* infection were significant correlates of HrQoL scores, with differential effects in high- and moderate-prevalence communities. The greatest differences were noted in the psychosocial domains of performance. We conclude that exposure to urogenital schistosomiasis has an overall detrimental effect on HrQoL at a level of 2–4% impairment. New implementation of better diagnostics for children is expected to refine our estimates of this association, as will follow-up studies of HrQoL following effective individual and community deworming.

## Introduction

Schistosomiasis remains a public challenge globally with 93% of the estimated 237 million infections occurring in Africa [1]. Its transmission is mainly influenced by exposures to environmental factors (contact with infested water, distance to infected water bodies), individual characteristics (treatment history, sex, and age) and socioeconomic factors (occupation and education) [2]–[5]. Schistosomiasis is rarely fatal but due to its recurring nature is manifested as a persistent chronic disorder in endemic areas, resulting in significant health burden [6]–[8]. It is estimated that people living in *Schistosoma -*endemic areas carry the infection one-third to one-half of their lives [9], yet they may only rarely exhibit the advanced morbidities that are classically associated with schistosomiasis, such as advanced hepatic fibrosis with portal hypertension (for *S. mansoni*), or bladder and kidney deformity, bladder cancer, or infertility (for *S. haematobium)* [7]. In reality, much of the negative health impact is due to less obvious or specific conditions such as anemia, undernutrition, abdominal pain, exercise intolerance, poor school performance, and lowered work capacity [7], [8], [10]. The non-specificity of chronic infection symptoms, manifested as these subtle morbidities, makes it difficult to accurately estimate the specific disease burden due to schistosomiasis. As pointed out by King and Bertino [11], the present disability-adjusted life year (DALY) system of the World Bank and the World Health Organization (WHO) [12] not only ignores these morbidities, but also disregards the pervasiveness of co-morbidities, including polyparasitism, in *Schistosoma-*endemic areas.

While the debate on DALY estimates of disease burden persists, attention is turning to the use of patient-reported outcomes, such as health-related quality of life (HrQoL) [6], [7], [11], [13]. Use of quality of life (QoL) assessment tools in evaluating schistosomiasis and other neglected tropical disease (NTD) burden is gaining greater standing [13]–[16]. Quality of Life is defined as an individual's perception of one's position in life in the context of culture and value systems, as well as in relation to one's goals, expectations, standards and concerns [17]. The World Health Organization defines health as being “not only the absence of disease and infirmity but also the presence of physical, mental, and social well-being” [18]. HrQoL therefore refers to the physical, psychological, and social scales of health seen in functional areas influenced by a person's experiences, beliefs, expectations, and perceptions. Measuring HrQoL is an important outcomes indicator in evaluating health-care interventions and treatments, in understanding the burden of disease, in identifying health inequalities, in allocating health resources, and in epidemiological studies and health surveys [19]. Unlike in other chronic conditions such cancer and sickle cell disease where HrQoL tools have been widely used, for NTDs these tools have only been used to assess the burden of advanced schistosomiasis (using EQ-5D plus or WHO QoL-bref questionnaires) [13], [15], [20], soil-transmitted helminthes (using both EQ-5D and SF-12) [16], and echinococcosis (using SF-12) [21], and to assess the burden of polyparasitism in Cote d'Ivoire (using SF-36v2 questionnaire but only the physical scale) [14]. SF-12 has also been used to describe the impact of acute schistosomiasis on quality of life in a group of travelers returning from a luxury safari trip in Tanzania [22]. Results of most of these studies suggest that the burden of schistosomiasis has been consistently underestimated [13], [15], [21]. Moreover, in two of these studies by Jia and colleagues, by only targeting people with chronic schistosomiasis [13] or advanced schistosomiasis [15] it was not possible to contrast HrQoL findings among schistosomiasis patients to people living in same localities without schistosomiasis. Additionally all these studies used QoL tools that do not capture the changes in QoL that occur in different developmental stages of children, the most important epidemiological demographic for active *Schistosoma* infection [23].

The choice of the HrQoL tool depends mainly on the purpose (the health conditions being investigated) and the target population (the general population, adults only or children only) [24], [25]. Measurement of HrQoL in children is particularly difficult because of the need for different instruments in different age groups, and the need for instruments that accommodate the different cultures. Two types of HrQoL measures have been developed, *generic* and *condition-specific* instruments. Generic or non-categorical instruments typically include global or summary ratings of multiple scales or health profile approaches. In contrast, condition-specific measures of HrQoL address the challenges associated with a particular illness, such as cancer. Since our target population in this study was children aged 5–18 years, we decided to use the Pediatric Quality of Life Inventory (PedsQL). PedsQL is available in two generic instruments; one that comprises 23 items (PedsQL) with forms for adult (over 26 years), young adult (18–25 years), adolescent (13–18 years), child (8–12 years), young child (5–7 years) and toddler (2–4 years) and a shorter instrument with 15 items (PedsQL 4.0 SF15) which has forms for adolescent, child, young child, and toddler. Furthermore, there are PedsQL disease-specific modules including modules for arthritis, asthma, cerebral palsy, cardiac, diabetes, family impact, family information, oral health and transplant among others (http://www.pedsql.org/ [26]). The validity and reliability of the instrument have been confirmed as a population health measurement tool and in different child populations with chronic illnesses in descriptive and evaluative studies [27]–[30]. The PedsQL has been used in children with different debilitating conditions such as those associated with asthma, transplant recipients, attention-deficit hyperactivity disorder (ADHD) and neuromuscular disorders [31]–[34].

The present study evaluated health related quality of life (HrQoL) in children living in a *S. haematobium* endemic area in coastal Kenya, and determined the utility of the pediatric quality of life inventory short form (PedsQL 4.0 SF15) in assessing HrQoL. Additionally, we determined the impact of local transmission features and socioeconomic standing, which are considered potentially important modifiers of *S. haematobium*–related disease burden.

## Methods

### Ethics statement and eligibility criteria

Ethical clearance was obtained by the Institutional Review Board at the University Hospital Case Medical Center of Cleveland and the Ethical Review Committee of the Kenya Medical Research Institute (KEMRI). Children were eligible if they were residents of the area for at least two years, were between 5–18 years old, and had provided child assent and written parental consent.

### Study area and population

**Figure 1:**
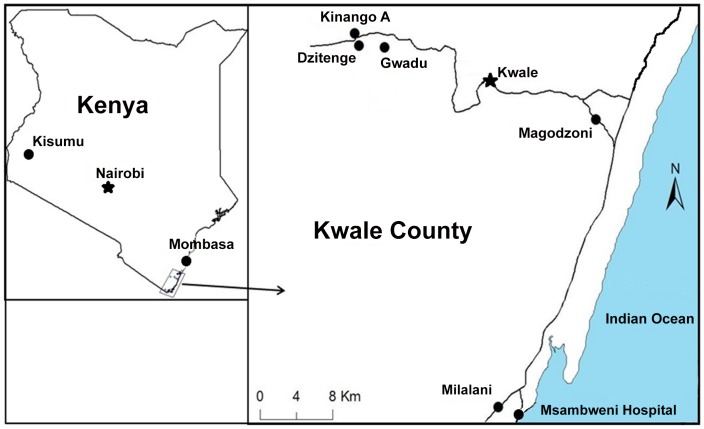
Map of the study area showing location of study villages in Coast Province, Kenya.

The study population comprised children aged 5–18 years old. who participated in both parasitological and nutritional studies in the five selected endemic rural villages (Milalani, Magadzoni, Gwadu, Dzitenge and Kinango A) in the three districts (Msambweni, Kinango, and Kwale) of Kwale County in Coast Province, Kenya [10], [35]–[37] (Figure 1). This sub-study was embedded in a larger study of the ecology of transmission of vector-borne parasitic infections (the ‘Polyparasitism Project’). This project enrolled participants through house-to-house demographic surveys in May–June 2009 for Milalani and in June, July, August and August–September 2010 for Gwadu, Dzitenge, Kinango A, and Magadzoni, respectively. Parasitological testing and anthropometric measurements were conducted simultaneously in July–August 2009 for Milalani, in October–November 2010 for Gwadu, Dzitenge, and Kinango A, and in April–May 2011 for Magadzoni. Treatment for any parasite infections detected during survey testing was provided immediately after the completion of each village survey. Due to logistical constraints, the PedsQL 4.0 SF15 questionnaires (see below) were administered at varying (3–16 month) intervals afterwards: in December 2010 for Milalani, in April–May 2011 for Gwadu, Dzitenge, and Kinango A, and in July 2011 for Magadzoni.

**Figure 2:**
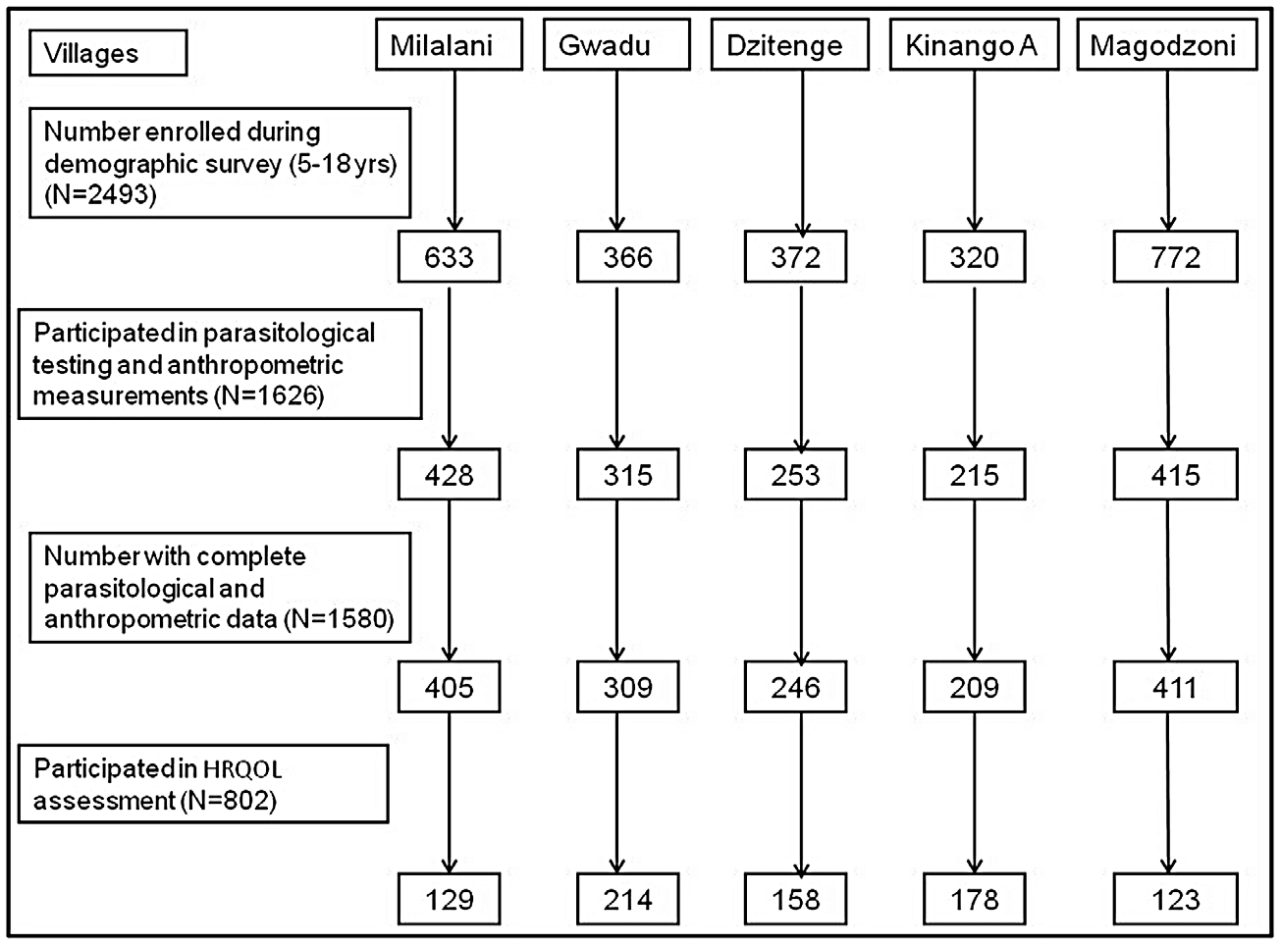
Flow chart of study participation. Numbers of children by village at enrolment, at parasitological and anthropometric testing, and at HrQoL assessment.

The numbers of participants per village at enrolment, at parasitological testing/anthropometric assessment, and at HrQoL assessment are detailed in Figure 2. All children with full parasitological and anthropometric results were eligible for inclusion in the study. For Milalani and Magadzoni villages, we randomly selected 92 children who had been *S. haematobium* egg-positive and 91 who had been *S. haematobium* egg-negative in each community for PedsQL 4.0 SF15 tool administration. For the other villages, we targeted all eligible children, irrespective of their initial egg-output status, for PedsQL 4.0 SF15 administration.

### Parasitological methods

Subjects submitted one midday sample for examination for *S. haematobium* infection. Ten-milliliter aliquots from well-mixed urine samples were subjected to standard Nucleopore filtration [38]. A single stool examination by Kato-Katz method [39] was used to identify infection by hookworm and other soil-transmitted helminths. All children included in this analysis provided finger prick blood for hemoglobin (Hb) measurement (Hemocue, Ängelholm, Sweden) [10]. Anemia was categorized according to WHO criteria by age and sex [40]– for ages <12 years, Hb<11.5 g/dL; for ages ≥12 years, Hb<12 g/dL; but for males ≥15 years, Hb<13 g/dL.

### Anthropometric measurement

Our standardization and measurement procedures used for anthropometric assessments have been detailed elsewhere (Bustinduy, *et al.*, [10]). The nutritional indicators; height-for-age (HAZ) and body-mass index (BMI)-for-age (BAZ) were computed using the World Health Organization's Anthro-Plus software for ages 5–19 years (WHO, Geneva, Switzerland) based on reference growth standards from the year 2006 [41], [42]. Stunting and wasting were defined for values≤−2 for HAZ and BAZ, respectively, according to WHO standards [43].

### HrQoL measurements

The PedsQL 4.0 SF 15 used in this study includes parallel child self-reports (age range 5–18 years) and parent proxy-reports. It differs from PedsQL 4.0 generic core scales instrument by the number of items in the total scale and in the various subscales. The PedsQL 4.0 SF15 consists of 5, 4, 3, and 3 for physical, emotional, social and school functioning respectively, making a total scale of 15 items whereas the PedsQL 4.0 generic core scale comprises of 8, 5, 5, and 5 for physical, emotional, social and school functioning, respectively, for a total of 23 items. Details are available at http://www.pedsql.org/about_pedsql.html.

The survey's preliminary instructions indicate to the subject that survey questions ask how much of a problem each item has been during the past one month. A five-point Likert-like response scale is used. The response scale for each item was “never” (0), “almost never” (1), “sometimes” (2), “often” (3), and “almost always” (4). Responses were transformed to 100, 75, 50, 25, and 0, respectively, resulting in a scale range of 0–100, with the higher number scores indicating better HrQoL.

Overall scores and sub-scale scores were computed as the sum of the items divided by the number of items answered (this accounts for missing data). If more than 50% of the items in the scale were missing, the scale score was not computed [44]. Two summaries and one overall score were computed. Of note, the physical health summary score (5 items) is the same as the physical functioning subscale. The psychosocial health summary score (10 items) is computed as the sum of items divided by the number of items answered in the emotional, social, and school functioning subscales. Total Scale Scores for child self-report and parent proxy-report are also presented.

In preparation for the study, two forward translations (English-Swahili) and one backward translation (Swahili-English) translations were done for the PedsQL 4.0 SF15. The approved final Swahili translations of the PedsQL 4.0 SF15 were first pre-tested on 23 young children (5–7 years old), 39 children (8–12 years old) and 36 teens and their parents from a neighboring village. These were all non-participants in the present study but similar to those included in this study. The illiteracy levels in the study area were estimated at about 60% and thus the PedsQL 4.0 SF15 instrument was interview administered [21], [45] for both children and their parents. All the PedsQL 4.0 SF15 questionnaires in this study were administered by two well-trained research assistants who were native speakers of Swahili, under the supervision of the first author.

### Statistical analysis

#### Biological, demographic, parasitological, anthropometric and socioeconomic data

Statistical analyses were conducted using both SPSS for Windows, version 19.0 (SPSS, Inc., Chicago, IL) and SAS for Windows, version 9 (SAS Institute Inc., Cary, North Carolina). For estimation of socioeconomic standing (SES) we used housing conditions and household ownership of selected assets to construct an asset index based on principal component analysis (PCA) [46]. Participating households were divided into two equal sized groups (low and higher socioeconomic standing) according to their scores from the principal components analysis. The assets included in the PCA were radio, bicycle, television, telephone, and land. Other indicators of SES included available household source(s) of cooking fuel, lighting, and drinking water, type of latrine owned and its distance from the house, and level of education of the household head.

Chi-square tests were used to test for differences in proportions, and the Mann-Whitney U test was used to assess differences in intensities of infection, age, and Hb. Analysis of variance (ANOVA) was used to compare infection intensity among villages.

### Analysis of HrQoL data

**Feasibility** of the PedsQL 4.0 SF15 generic version was determined from the average percentage of missing responses. The percentage of all possible item responses left unanswered was calculated for each subject on each single and summary scale and averaged over subjects.

**Utility** of the instruments in terms of distributional coverage overall and by subscale was evaluated by calculating the percentage of subscale-level average responses reaching the minimum (floor) or the maximum (ceiling) of the scoring scale. In QoL studies, floor and ceilings effects are used to evaluate the depth of a health problem being measured. If floor effects exist, it means the QoL tool is showing a lower than actual HrQoL, and if ceiling effects exist, QoL tools may be underestimating QoL or the magnitude of the problem being measured. Studies with small floor or ceiling effects (1–15%) are considered to meet acceptable measurement standards, whereas studies with moderate floor or ceiling effects (>15%) are considered less precise in measuring latent constructs at the extremes of the scale [25], [47].

**Internal consistency** is a measure of the extent to which items in a questionnaire (subscale or scale) are correlated (homogeneous), thus measuring the same concept. It is an important measurement property for questionnaires that intend to measure a single underlying concept (construct) by using multiple items such as PedsQL questionnaire. Cronbach's alpha coefficient was utilized to determine scale internal consistency reliability [48]. Scales with reliabilities of 0.70 or greater are recommended for comparing patient groups, while a reliability criterion of 0.90 is recommended for analyzing individual patient scores [49]. A low Cronbach's alpha indicates a lack of correlation between the items in a scale, which makes summarizing the items unjustified. A very high Cronbach's alpha (>0.95) indicates high correlations among the items in the scale, i.e., redundancy of one or more items [25]. Construct validity was determined utilizing the known-groups method, which compares scale scores across groups known to differ in the health construct being investigated. In our study the known groups are the children who tested positive and negative for *S. haematobium* egg output, who will henceforth be referred to as ‘*Sh* egg-positive’ and ‘*Sh* egg-negative’, respectively. Known groups validity was examined through a comparison of these egg-positive and egg-negative groups, of children from families of lower versus higher SES, of stunted versus non-stunted children, and those from high prevalence (high risk) villages versus moderate prevalence (lower risk) villages, using independent *t*-tests.

To complement statistical testing, effect sizes are presented to assist in the interpretation of the relative degree of between-group score differences by indexing these differences to within-group score variation [50], with lesser import if the between-group score difference is small relative to the within-group variation in scores. Effect size utilized in these analyses was calculated by taking the difference between the score means for either cases *vs.* controls, stunted *vs.* non-stunted children, children from low SES *vs.* high SES, or children from high-risk *vs.* moderate risk villages, divided by the pooled standard deviation of the egg-negative/high SES/not stunted/lower-risk village categories, as appropriate [51]. Effect sizes for differences in means are designated as small (0.20–0.49), medium (0.50–0.79), and large (≥0.80) in magnitude [52]. Agreement between child self-report and parent proxy-report was determined through 2-way mixed-effect model (absolute agreement, single measure) intraclass correlations [53]. Intraclass correlation results are generally interpreted as follows: ≤0.40, poor to fair agreement; 0.41–0.60, moderate agreement; 0.61–0.80, good agreement; and 0.81–1.00, excellent agreement [54].

### Multivariable modeling of HrQoL outcomes

Following our observation of significant differences in group-wise mean HrQoL scores in the analyses described above, our next objective was to determine the independent contributions of age, sex, village risk for schistosomiasis (high *vs.* moderate), *S. haematobium* infection, hookworm infection, anemia, SES, stunting, and wasting in nested models of HrQoL outcomes, for both total and psychosocial sub-scale scores. To do this, we used generalized multivariable linear modeling, adjusted for covariance at the village level using generalized estimating equation (GEE) technique (SPSS). Stepwise backward removal of non-significant variables was used to create ‘best fit’ parsimonious models based on Akaike information criteria (AIC) retaining explanatory variables with P-values<0.1. Significant multiply-adjusted parameter estimates are reported (with 95% CI and corresponding p-values) for covariates remaining in the final models.

## Results

### Study group characteristics

In total, 1580 children, aged 5–18 years old, from five villages participated in parasitological and anthropometric testing. Their mean age was 10.6±3.5 and 51% were female. The majority of the children in Milalani, Magadzoni, and Gwadu (the more rural villages) came from families of low SES while most children in Kinango A and Dzitenge (the more urban villages) came from families of higher SES (Table 1, Figure 1).

**Table 1:** Population demographic, anthropometric, and socioeconomic features, and distribution of *S. haematobium* egg positivity by village.

	Villages						
	Milalani (n = 405)	Magadzoni (n = 411)	Gwadu (n = 309)	Dzitenge (n = 246)	Kinango A (n = 209)	All Villages (n = 1580)	*P* value^a^
**Mean age (SD)**	10.9±3.5	10.9±3.5	10.6±3.6	10.3±3.3	10.2±3.3	10.6±3.5	**<0.05**
**% Female**	50.4	49.1	53.0	51.6	54.0	51.3	>0.5
**% Lower SES**	63.5	52.5	71.2	29.3	22.5	51.4	**<0.001**
***Sh+*** ** prevalence**	62.2	22.9	53.4	37.8	29.7	42.2	**<0.001**
***Sh+*** ** geometric mean intensity (eggs per 10 ml of urine)**	40.4	16.5	49.0	40.4	42.5	37.5	**<0.01**
**% Anemic** b	48.6	25.5	38.5	35.4	33.0	36.5	**<0.001**
**Mean hemoglobin, gm/dl (range)**	11.7 (4.6–16.4)	12.4 (4.3–17.2)	12.1 (5.2–17.5)	12.2 (6.8–16.5)	12.3 (6.6–17.4)	12.1 (4.3–17.5)	**<0.001**
**% Stunted** c	29.1	37.2	34.1	20.3	17.6	29.3	**<0.001**
**% Wasted** d	11.8	22.6	10.7	8.5	11.0	13.8	**<0.001**

Abbreviations: SD, standard deviation; SES, socioeconomic standing; Sh+, *Schistosoma haematobium* egg-positivity.

a P value refers to significance of differences among the villages by Mann-Whitney U test, ANOVA, or chi-square testing.

b Anemia based on WHO age-specific hemoglobin (Hb) criteria [40]: for ages <12 years, Hb<11.5 g/dl; for ages ≥12 years, Hb<12 g/dl; but for males ≥15 year, Hb<13 g/dl.

c Stunting: in height-for-age Z score (HAZ)≤−2.

d Wasting: BMI-for-age Z score (BAZ)≤−2.

### Parasitology

The overall *S. haematobium* infection prevalence was 42.2% (766/1580), similar for males and females, but significantly different by village (Table 1) and by age group (Table 2). School-age infection prevalence was significantly greater in Milalani, and Gwadu villages (here referred to as high-prevalence villages, according to WHO guidelines [55]) compared to Magadzoni, Dzitenge, and Kinango A villages (here referred to as moderate prevalence villages). *S. haematobium* infection intensity was highest in 8–12 year olds and lowest in 5–7 year olds (*P*<0.01, Table 2), and varied significantly by village (Table 1) with an overall geometric mean intensity of infection of 37.8 eggs/10 mL of urine. Males had significantly heavier infection than females (*P*<0.02). There were also significant inter-village and across-age group differences in the proportion of anemic children and in mean Hb levels (Tables 1 and 2).

**Table 2:** Characteristics of the study population by age group.

	5–7 years (n = 358)	8–12 years (n = 716)	13–18 years (n = 506)	P value^a^
**Mean age (SD)**	6.1±0.8	10.0±1.4	14.6±1.5	**–**
**% Female**	48.3	53.2	50.6	>0.2
**% Lower SES**	55.3	50.4	50.0	>0.2
***Sh+*** ** prevalence**	33.0	42.0	48.8	**<0.001**
***Sh+*** ** geometric mean intensity (eggs/10 ml of urine)**	28.7	48.7	31.2	**<0.05**
**% Anemic** b	36.9	33.5	40.5	**<0.05**
**Mean hemoglobin, g/dl (Range)**	11.9 (4.3–16.5)	12.1 (4.6–17.4)	12.4 (5.2–17.5)	**<0.001**
**% Stunted** c	17.3	30.2	36.6	**<0.001**
**% Wasted** d	7.0	14.4	17.8	**<0.001**

Abbreviations: SD, standard deviation; SES, socioeconomic standing; *Sh*+, *Schistosoma haematobium* egg-positivity.

a P value refers to significance of differences among the villages by Mann-Whitney U test, ANOVA, or chi-square testing.

b Anemia based on WHO age-specific hemoglobin (Hb) criteria [40]: for ages <12 years, Hb<11.5 g/dl; for ages ≥12 years, Hb<12 g/dl; but for males ≥15 years, Hb<13 g/dl.

c Stunting: in height-for-age Z score (HAZ)≤−2.

d Wasting: BMI-for-age Z score (BAZ)≤−2.

### Anthropometric outcomes

Many study children had either acute undernutrition, as measured by wasting (BAZ score ≤−2), chronic undernutrition, as measured by stunting prevalence (HAZ score ≤−2), or both. The highest malnutrition levels were recorded in villages closer to the coastline (Milalani, Magadzoni, and Gwadu) compared to the more inland villages (Dzitenge and Kinango A) (Table 1). Wasting and stunting were lowest in the 5–7 year age group compared to older age groups (Table 2). Significantly more males were stunted (56% *vs.* 44%, Χ^2^ = 9.2, P<0.01) or wasted (60% *vs.* 40%%, Χ^2^ = 14.8, P<0.001) as compared to females.

### HrQoL analysis

#### Participation

The PedsQL SF15 tool was administered to 835 children and 800 of their parents. Overall, missing responses were found for 33 children (4.0%) and 35 parents (4.4%), yielding 802 and 765 children and parents with complete HrQoL data, respectively (Table 3). The distribution of 802 children who had complete HrQoL data by village and age groups is shown in Figure 2.

**Table 3:** Feasibility and utility of PedsQL SF15 for children initially *S. haematobium* egg-positive or egg-negative^a^.

Scale	# items	Egg-positive (n = 352)			Egg-negative (n = 450)			Overall (n = 802)		
		% Missing	% Floor^b^	% Ceiling^b^	% Missing	% Floor	% Ceiling	% Missing	% Floor	% Ceiling
**Child self-report**										
Physical functioning	5	0.3	0.0	**71.0**	0.6	0.2	**71.6**	0.5	0.1	**71.3**
Emotional functioning	4	0.0	0.0	8.5	0.9	0.0	14.7	0.5	0.0	12.0
Social functioning	3	0.0	0.0	**27.6**	0.2	0.4	**34.7**	0.1	0.2	**31.5**
School functioning	3	3.3	1.1	**74.4**	2.8	1.3	**77.8**	3.0	1.2	**76.3**
Psychosocial score	10	3.3	0.0	5.1	3.6	0.0	9.6	3.5	0.0	7.6
Total scores	15	3.6	0.0	4.5	4.3	0.0	8.0	4.0	0.0	6.5
**Parent proxy-report**		**n = 333**			**n = 432**			**n = 765**		
Physical functioning	5	0.3	0.0	**78.1**	0.7	0.2	**74.5**	0.5	0.1	**76.1**
Emotional functioning	4	0.0	0.0	**20.1**	0.9	0.2	**25.9**	0.5	0.1	**23.4**
Social functioning	3	0.0	0.0	**35.4**	0.2	0.0	**45.1**	0.1	0.0	**40.9**
School functioning	3	4.8	0.0	**46.8**	2.0	0.2	**50.2**	3.3	0.1	**48.8**
Psychosocial score	10	4.8	0.0	12.6	3.1	0.0	**17.6**	3.9	0.0	**15.4**
Total scores	15	5.1	0.0	12.6	3.8	0.0	**15.7**	4.4	0.0	14.4

a Cases were those children initially found to have *S. haematobium* eggs on urine filtration during parasitological surveys. Controls were children who had been egg-negative. N.B. The PedsQL SF15 was administered 3–16 months after completion of testing and treatment for infection.

b % Floor/Ceiling = the percentage of scores at the extremes of the scaling range. Floor or ceiling effects in the range of 1–15% are acceptable while those >15% [**in BOLD**] are considered to provide less precise estimates.

#### Feasibility

Table 3 shows the missing responses, and the floor and ceiling effects noted in the PedsQL 4.0 SF15 administration in our study area. Missing values were found in both child self-report (range: 0.0–5.4%) and parent proxy-reports (range: 0.2–4.4%). The school function scale had the highest missing values for both self and parent proxy-reports (Table 3). Floor effects were found in both self and parent-proxy reports (range: 0.0–1.3). Influential ceiling effects (*i.e.*, >15%) were found in the physical, social, and school function scales in self report, with highest values noted for the school function scale. Relevant ceiling effects were found in all scales in the parent proxy report except for the total scores (Table 3).

#### Internal consistency of the questionnaire

The internal consistency/reliability alpha coefficients are presented for the PedsQL SF15 in Table 4. All the parent proxy-report scales exceeded the minimum internal consistency reliability standard (0.70) required for group comparisons for all ages. In child self-reports, reliability coefficients for most PedsQL SF15 scales approached or exceeded the recommended standard of 0.70 for group comparison (Table 4).

**Table 4:** Internal consistency reliability in self-report and parent proxy-report– Cronbach alpha statistics^a^ within different survey domains.

Scale	Young children (5–7 years)		Children (8–12 years)		Adolescent (13–18 years)		Overall	
	Cases^b^	Controls^b^	Cases	Controls	Cases	Controls	Cases	Controls
**Child self-report**	**n = 84**	**n = 109**	**n = 147**	**n = 200**	**n = 121**	**n = 141**	**n = 352**	**n = 450**
Physical functioning	0.86	0.89	0.86	0.87	0.83	0.87	0.85	0.87
Emotional functioning	0.79	0.81	0.76	0.82	0.74	0.80	0.76	0.81
Social functioning	0.83	0.85	0.79	0.82	0.76	0.80	0.79	0.82
School functioning	0.84	0.86	0.84	0.85	0.83	0.83	0.83	0.85
Psychosocial score	0.73	0.77	0.70	0.75	0.66	0.73	**0.69**	0.74
Total scores	0.72	0.76	**0.68**	0.73	**0.64**	0.71	**0.67**	0.73
**Parent proxy-report**	**n = 80**	**n = 105**	**n = 139**	**n = 192**	**n = 114**	**n = 135**	**n = 333**	**n = 432**
Physical functioning	0.88	0.90	0.87	0.90	0.87	0.87	0.87	0.89
Emotional functioning	0.84	0.80	0.78	0.86	0.83	0.84	0.81	0.84
Social functioning	0.88	0.82	0.80	0.87	0.87	0.86	0.85	0.86
School functioning	0.86	0.87	0.85	0.87	0.86	0.86	0.86	0.87
Psychosocial score	0.79	0.77	0.72	0.80	0.78	0.78	0.76	0.79
Total scores	0.78	0.75	0.71	0.79	0.77	0.77	0.75	0.78

a Cronbach alpha values ≥0.70 are recommended for comparing patient groups, and ≥0.90 are recommended for analyzing individual patient scores [49].

b Cases were those children initially found to have *S. haematobium* eggs on urine filtration during parasitological surveys. Controls were children who had been egg-negative. N.B. The PedsQL SF15 was administered after testing and treatment for infection.

#### Parent-child concordance

Table 5 presents the intraclass correlations (ICCs) between child self-reports and parent proxy-reports of the PedsQL SF15 scales. Fair to good agreement was found in the generic core scales of both the *S. haematobium* egg-positive and egg-negative (control) groups. ICCs were generally higher in the control group. Lower correlation values were obtained in the social function scales across all age groups, and in the physical scale in 5–7 year olds. Children and their parent proxies consistently showed good to excellent agreement for school and psychosocial scales across all age groups.

**Table 5:** Agreement between self-report and parent proxy-report PedsQL SF15 score scales.

	Young children (5–7 years)	Children (8–12 years)	Teens (13–18 years)	All ages
***Sh*** a **egg-positive**				
Physical functioning	0.09	**0.51**	**0.55**	**0.47**
Emotional functioning	**0.41**	0.23	0.38	0.34
Social functioning	0.19	0.20	0.13	0.18
School functioning	**0.56**	**0.49**	**0.58**	**0.54**
Psychosocial score	**0.93**	**0.90**	**0.92**	**0.92**
Total scores	0.30	0.30	**0.50**	0.37
***Sh*** ** egg-negative**				
Physical functioning	0.37	**0.49**	0.40	**0.44**
Emotional functioning	0.40	0.40	0.34	**0.41**
Social functioning	0.37	0.28	**0.47**	0.39
School functioning	**0.64**	**0.60**	**0.63**	**0.65**
Psychosocial score	**0.94**	**0.93**	**0.95**	**0.94**
Total scores	**0.57**	**0.47**	**0.52**	**0.55**

Inter-class correlation (ICC) values for survey results are considered as poor to fair agreement (≤0.40), moderate agreement (0.41–0.60), good agreement (0.61–0.80) or excellent agreement (0.81–1.00) [54]. **Bold face** indicates moderate or better agreement between child and parent-proxy.

a Abbreviation: Sh, *Schistosoma haematobium.*

#### HrQoL outcomes according to location, infection status, SES, and known morbidities

**Figure 3:**
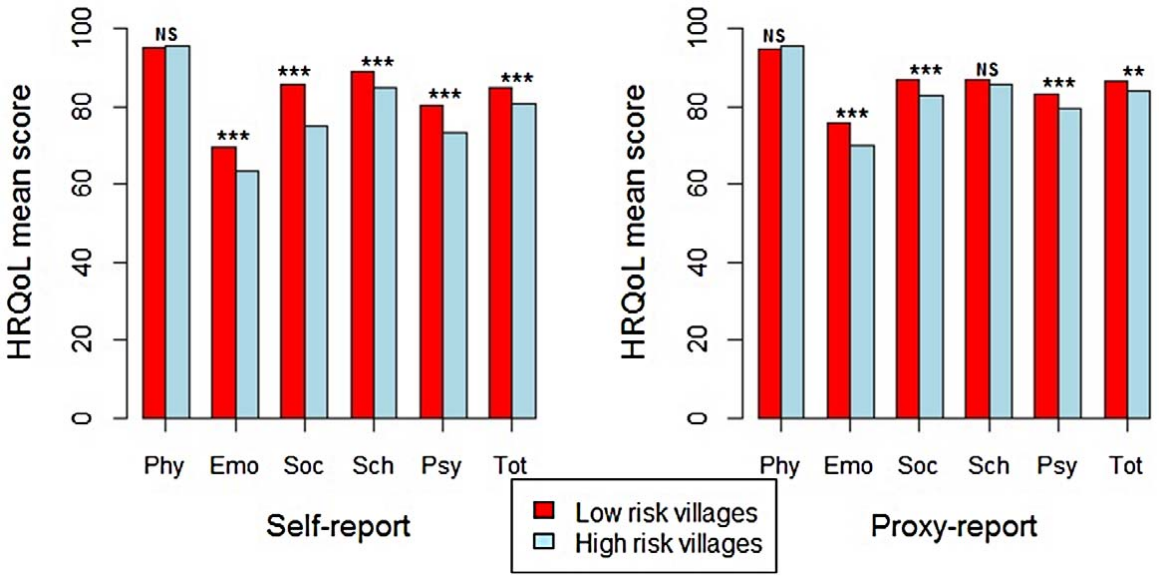
PedsQL SF15 score scales contrasting high and moderate risk villages for self and proxy reports. Abbreviations: Phy-Physical; Emo-Emotional; Soc-Social; Sch-School; Psy-Psychosocial, Tot-Total. NS-Not significant; **P<0.001; ***P<0.0001.

To assess the impact of exposure to urogenital schistosomiasis, we examined differences in observed PedsQL SF15 outcomes on several levels—high-prevalence *vs.* moderate-prevalence villages, *S. haematobium* egg-positive *vs.* egg-negative individuals, and according to cofactors likely to influence HrQoL (presence or absence of lower SES, anemia, or undernutrition). The most striking finding was the significantly higher HrQoL scores reported by both children and parent residing in moderate risk villages as compared to those from high risk villages (Figure 3). Furthermore, within the moderate risk villages, when comparing PedsQL SF15 scores, egg-negative children had significantly higher HrQoL in all scales except physical and school scales (for both child-self and parent-proxy reports), with effect sizes mostly in the medium range (Table 6). Of note, the PedsQL SF15 egg-positive *vs.* egg-negative score differences within the high risk villages were small and not significant, and mostly in the opposite direction for most scales (Table 6).

**Table 6:** PedsQL SF15 score scales for children and parents within high and moderate prevalence villages.

	High Prevalence			Moderate Prevalence		
	*Sh* egg +ve^a^	*Sh* egg −ve^a^	Effect size	*Sh* egg +ve	*Sh* egg −ve	Effect size^b^
**Scale**	Mean (SD)	Mean (SD)		Mean (SD)	Mean (SD)	
**Child self-report**	n = 241	n = 260		n = 111	n = 190	
Physical functioning	96.0 (9.5)	95.1 (11.2)	−0.08	93.6 (13.8)	95.7 (12.2)	0.20
Emotional functioning	63.7 (19.5)	63.2 (19.5)	−0.04	66.7 (17.8)	71.3 (20.5)*	0.44
Social functioning	75.2 (20.4)	75.0 (21.3)	−0.02	83.2 (14.1)	86.9 (14.6)*	0.35
School functioning	84.8 (17.3)	84.5 (19.9)	−0.03	88.7 (15.8)	88.8 (15.5)	0.09
Psychosocial score	73.5 (13.5)	73.1 (14.6)	−0.04	78.3 (11.2)	81.3 (12.8)*	0.29
Total scores	81.0 (9.7)	80.4 (10.9)	−0.05	83.4 (9.7)	86.1 (10.5)*	0.26
**Parent proxy-report**	n = 225	n = 249		n = 108	n = 183	
Physical functioning	96.1 (11.7)	95.2 (12.0)	−0.07	94.1 (14.6)	94.9 (13.2)	0.07
Emotional functioning	70.5 (23.8)	69.6 (25.1)	−0.07	72.1 (19.4)	77.7 (17.9)*	0.51
Social functioning	82.3 (17.1)	83.5 (17.8)	0.10	84.9 (13.9)	88.3 (14.4)*	0.31
School functioning	84.9 (20.1)	86.2 (19.5)	0.10	86.8 (17.6)	86.7 (19.3)	−0.09
Psychosocial score	79.3 (14.7)	79.7 (15.6)	0.17	81.3 (11.7)	84.2 (12.9)*	0.27
Total scores	84.3 (11.7)	84.2 (12.4)	−0.06	84.9 (10.9)	87.3 (10.9)	0.22

a Sh egg +ve were those children initially found to have *S. haematobium* eggs on urine filtration during parasitological surveys. *Sh* egg −ve were children who had been egg-negative. N.B. The PedsQL SF15 was administered after testing and treatment for infection.

b Effect size = (difference between cases and controls)/SD of controls. Effect sizes are designated as small (.20), medium (.50), and large (.80).

* p<0.05 (independent samples t-test).

For all PedsQL SF15 scales (except physical functioning in parent proxy-report) children who tested negative for *S. haematobium* egg output and their parents reported better HrQoL than those who tested egg-positive during parasitological examination (Table 7). However, for the overall combined village data, where the largest effect size observed for the social subscale (0.21), none of these group-wise differences in child self-report was statistically significant. Parents of egg-negative children reported better HrQoL scores for their children compared to parents of egg-positive cases for all subscales, except in the physical subscales. These parental HrQoL scores were only statistically different for the social scale, and effect sizes were generally of small magnitude.

**Table 7:** PedsQL SF15 scores for study participants stratified according to *S. haematobium* egg-positive *vs.* egg-negative status.

Scale	# items	*Sh* egg-positive^a^			*Sh* egg-negative^a^			Difference	Effect Size^b^	*t* score	P value^c^
		n	Mean	SD	n	Mean	SD				
**Child self-report**											
Physical functioning	5	352	95.3	11.1	450	95.4	11.6	0.1	0.09	0.07	>0.5
Emotional functioning	4	352	64.7	19.0	450	66.6	20.3	1.9	0.17	1.39	>0.1
Social functioning	3	352	77.7	19.0	450	80.0	19.7	2.3	0.21	1.65	>0.05
School functioning	3	352	88.3	22.4	450	89.0	22.6	0.7	0.06	0.45	>0.5
Psychosocial score	10	352	76.1	12.7	450	77.7	14.2	1.6	0.14	1.58	>0.1
Total scores	15	352	81.8	9.8	450	82.8	11.1	1.0	0.09	1.43	>0.1
**Parent proxy-report**											
Physical functioning	5	333	95.4	12.7	432	95.1	12.5	−0.3	−0.03	−0.41	>0.5
Emotional functioning	4	333	71.1	22.5	432	73.0	22.6	1.9	0.16	1.18	>0.2
Social functioning	3	333	83.2	16.2	432	85.5	16.6	2.3	0.20	1.97	**<0.05**
School functioning	3	333	85.5	19.3	432	86.4	19.4	0.9	0.08	0.63	>0.5
Psychosocial score	10	333	79.9	13.8	432	81.6	14.7	1.7	0.14	1.66	>0.1
Total scores	15	333	84.5	11.5	432	85.5	11.8	1.0	0.09	1.22	>0.2

a Sh egg-positive were those children initially found to have *S. haematobium* eggs on urine filtration during parasitological surveys. *Sh* egg –negative were children who had been egg-negative. N.B. The PedsQL SF15 was administered after testing and treatment for infection. (n = 802 for child self-report and 765 for parent proxy report).

b Effect size = (difference between cases and controls)/SD of controls. Effect sizes are typically designated as small (0.20–0.49), medium (0.50–0.79), or large (≥0.80).

c *P*<0.05 (independent sample t-test).

For stunting, all scales reported lower quality of life in stunted children, with significant differences in psychosocial and total scales in self-reports, and in the school functioning scale in the proxy-report (see Supporting Information, Table S1). In both child self and parent proxy reports, lower SES was significantly associated with lower HrQoL in all scales except the physical functioning, with effect sizes mostly in medium to high range (see Supporting Information, Table S2). Within the low SES group, when comparisons were made between egg-positive and egg-negative children, egg-positives reported lower HrQoL compared to controls. However, when similar comparisons were made within the higher SES group, the differences were very small and inconsistent, with some scales reporting higher HrQoL among egg-positive children (data not shown).

Given the group-wise differences observed above, we next used a Generalized Estimating Equation (GEE) multivariable analysis to account for possible confounding and/or effect modification of score outcomes caused by varied distribution of subject classes across villages and infection status groups. As noted in Tables 8 and 9, HrQoL total scores and psychosocial sub-scores were significantly lower in high-prevalence villages, even with adjustment for SES, sex, and/or age. Not shown, hookworm infection or intensity did not have a significant effect on Peds QL scores when adjusted for other factors in the model. Variable interaction terms for village type were highly significant, leading to a stratified village type analysis also presented in Tables 8 and 9. In high-prevalence villages, lower SES, and stunting were significantly associated with lower total and psychosocial scores, whereas wasting and *S. haematobium* egg-positivity were associated with higher scores. In moderate-prevalence villages, *S. haematobium* egg positivity was the sole significant correlate of total HrQoL score, reducing it by an estimated 2.1%. For the psychosocial sub-scale, egg-positivity contributed on a similar level (−2.4%) to a lower score, while being in the 8–12 year old age group was associated with a significantly slightly better score (+0.8%).

**Table 8:** Multivariable GEE modeling of self-reported total HrQoL scores adjusting for measured covariates^a^.

	Combined villages		High *Sh* prevalence villages		Moderate *Sh* prevalence villages	
Variable	Parameter Estimate (95% CI)	P value	Parameter Estimate (95% CI)	P value	Parameter Estimate (95% CI)	P value
High prevalence village	**−4.0 (−4.9, −3.2)**	**<0.001**	–	–	–	–
Sex = Female	**−0.18 (−3.2, −0.04)**	**<0.05**	–	–	–	–
Lower SES^b^	**−2.0 (−4.0, −0.06)**	**<0.05**	**−4.1 (−5.8, −2.4)**	**<0.001**	–	–
Stunting^c^	−1.2 (−2.6, 0.12)	>0.05	**−3.1 (−4.2, −2.0)**	**<0.001**	–	–
Wasting^d^	–	–	**+3.1 (2.6, 3.6)**	**<0.001**	–	–
*Sh* egg-positive on initial survey	–	–	**+2.4 (0.2, 4.6)**	**<0.05**	**−2.1 (−3.9, −0.28)**	**<0.05**

In each case, the initial model of PedsQL SF15 score included the following explanatory variables: sex, age group, village type (high- vs. lower-endemicity), socioeconomic standing, current *Schistosoma* infection, current hookworm infection, anemia, presence of growth stunting, and nutritional wasting. Generalized multivariable linear modeling, adjusted for covariance at the village level using GEE estimation (SPSS) used stepwise backward removal of non-significant variables to create ‘best fit’ parsimonious models (based on information criteria) retaining explanatory variables with P-values<0.1. Multiply-adjusted parameter estimates are reported (with 95% CI and corresponding P-values) for covariates remaining in the final models. Scale for the HrQoL scale output variable was set at 100, so that the parameter estimates reflect percentage changes in overall HrQoL as estimated by the PedsQL instrument.

a Abbreviations: GEE, Generalized Estimating Equations; HrQoL, Health-related quality of life; Sh, *Schistosoma haematobium*; CI, Confidence Interval; SES, socio-economic standing.

b Reference group for comparison was top 50% SES.

c Stunting: height-for-age Z score (HAZ) ≤−2.

d Wasting: BMI-for-age Z score (BAZ) ≤−2.

**Table 9:** Multivariable GEE modeling of HrQoL psychosocial scores adjusting for measured covariates^a^.

	Combined villages		High *Sh* prevalence villages		Moderate *Sh* prevalence villages	
Variable	Parameter Estimate (95% CI)	P value	Parameter Estimate (95% CI)	P value	Parameter Estimate (95% CI)	P value
High prevalence village	**−5.7 (−6.7, −5.0)**	**<0.001**	–	–	–	–
Lower SES^b^	**−2.9 (−5.0, −0.9)**	**<0.01**	**−5.0 (−6.5, −3.5)**	**<0.001**	–	–
Stunting^c^	−1.4 (−2.9, 0.6)	>0.05	**−3.7 (−6.7, −0.7)**	**<0.05**	–	–
Wasting^d^			**+3.9 (3.3, 4.5)**	**<0.001**	–	–
*Sh* egg-positive on initial survey			+2.6 (−0.5, 5.7)	>0.05	−2.4 (−5.6, 0.7)	>0.1
Age 8–12^e^	**+0.7 (0.59, 0.83)**	**<0.001**	–	–	**+0.8 (0.07, 1.5)**	**<0.05**
Age 13–18^e^	–	–	**+1.2 (0.4, 2.0)**	**<0.01**	–	–

In each case, the initial model of PedsQL SF15 score included the following explanatory variables: sex, age group, village type (high- vs. lower-endemicity), socioeconomic standing, current *Schistosoma* infection, current hookworm infection, anemia, presence of growth stunting, and nutritional wasting. Generalized multivariable linear modeling, adjusted for covariance at the village level using GEE estimation (SPSS) used stepwise backward removal of non-significant variables to create ‘best fit’ parsimonious models (based on information criteria) retaining explanatory variables with P-values<0.1. Multiply-adjusted parameter estimates are reported (with 95% CI and corresponding P-values) for covariates remaining in the final models. Scale for the HrQoL scale output variable was set at 100, so that the parameter estimates reflect percentage changes in overall HrQoL as estimated by the PedsQL instrument.

a Abbreviations: GEE, Generalized Estimating Equations; HrQoL, Health-related quality of life; Sh, *Schistosoma haematobium*; CI, Confidence Interval; SES, socio-economic standing.

b Reference group for comparison was top 50% SES.

c Stunting: height-for-age Z score (HAZ) ≤−2.

d Wasting: BMI-for-age Z score (BAZ) ≤−2.

e Reference group was 5–7 year olds.

## Discussion

This is a first attempt to measure self-rated multidimensional HrQoL related to urogenital schistosomiasis in children. In our initial analysis, we observed a clear trend toward lower HrQoL in all measurement scales (except physical scale) among children with *S. haematobium* egg-positive status, but the differences were not significant. This trend was confirmed in parent-proxy reports, which indicated the same trend in all performance scales, and for the social scale, parents of egg-positive children did report significantly lower HrQoL scores than parents of egg-negative children. Significant differences were observed in other group-wise analysis, indicating lower HrQoL for children resident in high-*S. haematobium* prevalence villages as compared to those living in moderate-prevalence villages, and significantly lower HrQoL for children with growth stunting.

Previous investigations described strong associations between growth stunting and low intensity *Schistosoma* infections [6], [56]–[58]; thus the joint association of lower HrQoL with local prevalence and stunting was an expected finding. Our results are in contrast to recent studies of other NTDs that suggest no clear differences in HrQoL between infected and non-infected health states [14], [16]. Increasing severity of intestinal schistosomiasis, whether caused by *S. japonicum* in P.R. China [13], [15] or by *S. mansoni* in Egypt [20], has been associated with significantly greater reductions in self-reported quality of life scores. It is possible that inclusion of a larger range of age-groups, and patients with more advanced complications of *S. haematobium* would have resulted in more pronounced differences in our observed outcomes. We found significant associations between either i) lower socio-economic status, or ii) residence in high risk villages, with lower PedsQL 4.0 SF15 scores across all scales, except for physical and school functional scales. Socio-economic status is known to affect the risk of *Schistosoma* infection by either limiting water-use options and/or access to health care, or through other poverty-related factors [11], [57], [59], [60].

In our study, the initial lack of significant differences in HrQoL between children with and without egg output could be attributed to confounding by the distribution of low SES and undernutrition across the village levels. Our multivariable analysis indicated a strong village-type effect (with high-prevalence status having significantly lower HrQoL scores), but with significant interaction between village type and all other covariates tested. A further stratified analysis indicated that *S. haematobium* egg positivity was significantly associated with reduced HrQoL in moderate prevalence villages, whereas in high-prevalence villages, it was associated with higher HrQoL. This difference is likely to reflect issues with the imperfect sensitivity of our standard parasitological diagnosis of urogenital schistosomiasis (urine egg output [61]), and covariation between age- and SES-related infection risk. In addition, for schistosomiasis and other neglected tropical diseases, the time from the onset of active infection to the onset of clinical disease may be protracted. Also, a single urine exam may miss 20–30% of active infections that would be found by repeated urine exams [61]. Serological testing indicates that among children in high-prevalence villages, active or recent *Schistosoma* infection prevalence is close to 100% [23]. Light intensity infections are more easily missed in parasitological testing. However, these can still result in significant inflammation-mediated morbidity. We believe that in high-prevalence villages, infection status is already saturated, so that egg-positivity reflects mainly those with heavier infections. In the high-prevalence setting, this sub-group includes older children with greater mobility [62], who may therefore have better scores on the PedsQL scales. In moderate prevalence villages, where egg testing is more likely to reliably distinguish infected and uninfected children, *S. haematobium* egg-positivity was significantly associated with lower HrQoL scores. Overall, uncertainty (negative study bias) about the status of egg-negative controls may explain why we observed significant ‘infection-related’ differences in moderate-risk villages but not in high-risk villages. Finally, because PedsQL 4.0 SF15 questionnaire administration was not administered at the same time as the parasitological assessment, some of the ‘controls’ identified during parasitological assessment may have turned into ‘cases’ (i.e., those who were now or formerly *S. haematobium* infected) by the time HrQoL assessment was done. Because treatment was given before administration of the Peds QL survey, acute impacts of active infection may have gone missed, and these may have had an impact on our study findings. We recommend that similar studies in future carry both assessments concurrently, and include supplemental serologies (circulating antigen testing, anti-Schistosoma IgG4), in order to minimize the misidentification of the true infection status of ‘cases’ and ‘controls’.

The mode of administration of the PedsQL 4.0 SF15 questionnaire was interviewer-based; thus, the high response rate was not surprising. However, it may have contributed to the lack clear differences between the cases and controls, because interviewer-based modes of questionnaire administration suffer from a well- recognized problem of limited willingness to acknowledge a problem [24]. Since all interviews across all villages were conducted by two trained research assistants using one-to-one administration, we believe the interviewer effect was not significant. For the study children, inclusion of a parent-proxy survey, in addition to the direct patient survey, was considered to add external evidence of the validity of the answers we obtained from the children.

Floor effects were largely absent in the study population, except in the school scale where negligible (<1.3%) floor effects were observed, especially among controls. On the other hand, substantial ceiling effects were evident in almost all scales, and were more prominent in physical and school scales particularly among controls (*Sh* egg - negative). This means that the PedsQL 4.0 SF15 questionnaire likely underestimated HrQoL, especially for children who tested negative for *S. haematobium* egg output. While ceiling effects are a common phenomenon, they restrict the ability of the HrQoL tool to detect change or describe health above the average in more healthy populations [16], [63]–[65]

We consider the PedsQL to be a suitable tool for assessing quality of life in children with schistosomiasis. There is evidence that its reliabilities were high (alphas generally ≥0.70), floor effects were acceptable and identification of children from both low SES and ‘high risk’ villages was valid. PedsQL 4.0 SF15 was an effective tool for measuring quality of life in children living in schistosomiasis- (and likely other neglected tropical disease-) endemic areas. The clear capability of PedsQL 4.0 SF15 tool to identify geographical areas with different transmission intensities and SES groups is particularly important in schistosomiasis control. In large scale schistosomiasis control programs, its practicability (administered within 5 minutes) means it can appropriately be used to rapidly tease out high transmission localities for further adjustment. However, further research is needed, especially on its reproducibility and responsiveness (ability to detect clinically important changes over time) in relation to schistosomiasis. The difficulties in measuring physical and school health illustrated here also point to the need for further research and the development of a schistosomiasis specific PedsQL tool to enhance assessment of *Schistosoma* infection-related health impact.

## Supporting Information

Checklist S1 **STROBE checklist.**
(DOC)

Table S1 **PedsQL SF15 score scales for stunted children and non-stunted children.**
(DOCX)

Table S2 **PedsQL SF15 score scales for children from lower and higher socioeconomic standing.**
(DOCX)
