# Supplementary material for: Evaluation of the Health-related Quality of Life of Children in Schistosoma haematobium-endemic Communities in Kenya: A Cross-sectional Study
Source: PLoS Negl Trop Dis. 2013 Mar 7;7(3):e2106. doi: 10.1371/journal.pntd.0002106 (PMC3591318; doi:10.1371/journal.pntd.0002106)
Supplement: Table S1 — PedsQL SF15 score scales for stunted children and non-stunted children. (DOCX) [file pntd.0002106.s002.docx]

**Table S1: PedsQL SF15 score scales for stunted children and non-stunted children**

| **Scale** | **# items** | **Stunted children** | | | **Non-stunted children** | | | **Diffe-rence** | **Effect Size** | ***t* score** | **P value** |
| --- | --- | --- | --- | --- | --- | --- | --- | --- | --- | --- | --- |
|  |  | **n** | **Mean** | **SD** | **n** | **Mean** | **SD** |  |  |  |  |
| **Child self-report** | | | | | | | | | | | |
| Physical functioning | 5 | 236 | 94.6 | 10.8 | 566 | 95.6 | 11.6 | 1.0 | 0.09 | 1.11 | 0.27 |
| Emotional functioning | 4 | 236 | 63.9 | 19.7 | 566 | 66.5 | 19.7 | 2.6 | 0.25 | 1.71 | 0.09 |
| Social functioning | 3 | 236 | 77.8 | 19.6 | 566 | 79.5 | 19.6 | 1.7 | 0.16 | 1.13 | 0.26 |
| School functioning | 3 | 236 | 87.4 | 24.2 | 566 | 89.2 | 21.8 | 1.8 | 0.17 | 1.00 | 0.32 |
| Psychosocial score | 10 | 236 | 74.2 | 14.0 | 566 | 76.6 | 13.7 | 2.4 | 0.23 | 2.23 | 0.02* |
| Total scores | 15 | 236 | 81.0 | 10.3 | 566 | 82.9 | 10.6 | 1.9 | 0.18 | 2.35 | 0.02* |
| **Parent proxy-report** | | | | | | | | | | | |
| Physical functioning | 5 | 217 | 94.7 | 11.6 | 548 | 95.4 | 13.0 | 0.7 | 0.06 | 0.71 | 0.48 |
| Emotional functioning | 4 | 217 | 71.4 | 22.5 | 548 | 72.5 | 22.6 | 1.1 | 0.09 | 0.61 | 0.54 |
| Social functioning | 3 | 217 | 84.1 | 18.0 | 548 | 84.6 | 15.8 | 0.5 | 0.04 | 0.39 | 0.70 |
| School functioning | 3 | 217 | 83.6 | 20.6 | 548 | 87.0 | 18.8 | 3.4 | 0.29 | 2.16 | 0.03* |
| Psychosocial score | 10 | 217 | 78.9 | 15.6 | 548 | 80.5 | 14.4 | 1.6 | 0.14 | 1.35 | 0.18 |
| Total scores | 15 | 217 | 84.2 | 11.9 | 548 | 85.5 | 11.6 | 1.3 | 0.11 | 1.38 | 0.17 |

Effect size = (difference between stunted and not stunted respondents)/SD of not stunted respondents.

Effect sizes are designated as small (.20), medium (.50), and large (.80).

*p < .05 (independent samples t-test).
