# Supplementary material for: Evaluation of the Health-related Quality of Life of Children in Schistosoma haematobium-endemic Communities in Kenya: A Cross-sectional Study
Source: PLoS Negl Trop Dis. 2013 Mar 7;7(3):e2106. doi: 10.1371/journal.pntd.0002106 (PMC3591318; doi:10.1371/journal.pntd.0002106)
Supplement: Table S2 — PedsQL SF15 score scales for children from lower and higher socioeconomic standing. (DOCX) [file pntd.0002106.s003.docx]

**Table S2: PedsQL SF15 score scales for children from lower and higher socioeconomic standing**

| **Scale** | **# items** | **Children from low SES families** | | | **Children from higher SES families** | | | **Diffe-rence** | **Effect Size** | ***t* score** | **P value** |
| --- | --- | --- | --- | --- | --- | --- | --- | --- | --- | --- | --- |
|  |  | **n** | **Mean** | **SD** | **n** | **Mean** | **SD** |  |  |  |  |
| **Child self-report** | | | | | | | | | | | |
| Physical functioning | 5 | 352 | 94.7 | 11.6 | 450 | 95.9 | 11.1 | 1.2 | 0.12 | -1.51 | 0.13 |
| Emotional functioning | 4 | 352 | 62.3 | 19.4 | 450 | 68.9 | 19.6 | 6.6 | 0.64 | -4.78 | 0.00* |
| Social functioning | 3 | 352 | 75.6 | 19.9 | 450 | 82.1 | 18.4 | 6.5 | 0.63 | -4.83 | 0.00* |
| School functioning | 3 | 352 | 85.4 | 25.2 | 450 | 91.7 | 19.2 | 6.3 | 0.61 | -3.94 | 0.00* |
| Psychosocial score | 10 | 352 | 74.1 | 13.6 | 450 | 79.6 | 13.0 | 5.5 | 0.53 | -5.84 | 0.00* |
| Total scores | 15 | 352 | 80.2 | 10.3 | 450 | 84.3 | 10.3 | 1.5 | 0.40 | -5.67 | 0.00* |
| **Parent proxy-report** | | | | | | | | | | | |
| Physical functioning | 5 | 357 | 94.7 | 12.9 | 408 | 95.7 | 12.3 | 1.0 | 0.09 | -1.03 | 0.30 |
| Emotional functioning | 4 | 357 | 69.4 | 24.5 | 408 | 74.6 | 20.5 | 5.2 | 0.45 | -3.20 | 0.00* |
| Social functioning | 3 | 357 | 82.8 | 17.0 | 408 | 86.0 | 15.9 | 3.2 | 0.27 | -2.67 | 0.01* |
| School functioning | 3 | 357 | 84.4 | 20.8 | 408 | 87.5 | 17.9 | 3.1 | 0.27 | -2.16 | 0.03* |
| Psychosocial score | 10 | 357 | 78.9 | 15.7 | 408 | 82.7 | 12.8 | 3.8 | 0.33 | -3.70 | 0.00* |
| Total scores | 15 | 357 | 83.5 | 12.5 | 408 | 86. 5 | 10.8 | 3.0 | 0.26 | -3.50 | 0.00* |

Effect size = (difference between respondents from low and high SES families)/SD of respondents from high SES families.

Effect sizes are designated as small (.20), medium (.50), and large (.80).

*p < .05 (independent samples t-test).
